# Variations in leaf water status and drought tolerance of dominant tree species growing in multi-aged tropical forests in Thailand

**DOI:** 10.1038/s41598-022-10988-1

**Published:** 2022-04-27

**Authors:** Weerapong Unawong, Siriphong Yaemphum, Anuttara Nathalang, Yajun Chen, Jean-Christophe Domec, Pantana Tor-ngern

**Affiliations:** 1 Center of Excellence on Hazardous Substance Management, Chulalongkorn University, Bangkok, 10330 Thailand; 2 Graduate School, Chulalongkorn University, Bangkok, 10330 Thailand; 3 National Biobank of Thailand, National Science and Technology Development Agency, Pathum Thani, 12120 Thailand; 4 CAS Key Laboratory of Tropical Forest Ecology, Xishuangbanna Tropical Botanical Garden, Chinese Academy of Sciences, Menglun, Mengla, 666303 Yunnan China; 5 Yuanjiang Savanna Ecosystem Research Station, Xishuangbanna Tropical Botanical Garden, Chinese Academy of Sciences, Yuanjiang, 653300 Yunnan China; 6 Bordeaux Sciences AGRO, UMR 1391 ISPA INRA, 1 Cours du général de Gaulle 33175, Gradignan Cedex, France; 7 Nicholas School of the Environment and Earth Sciences, Duke University, Durham, NC 27708 USA; 8 Department of Environmental Science, Faculty of Science, Chulalongkorn University, Bangkok, 10330 Thailand

**Keywords:** Ecology, Plant sciences, Ecology, Environmental sciences

## Abstract

Large-scale abandoned agricultural areas in Southeast Asia resulted in patches of forests of multiple successions and characteristics, challenging the study of their responses to environmental changes, especially under climatic water stress. Here, we investigated seasonal variation in leaf water status and drought tolerance of dominant tree species in three multi-aged tropical forests, ranging from 5 to > 200 years old, with contrasting soil moisture in Thailand. Seasonal variation in leaf water status differed among the forests with trees in young and intermediate sites demonstrating larger differences between seasons than the old-growth forest. Although vulnerability to embolism curves revealed that trees in old-growth forest were potentially more sensitive to declining leaf water status than others, they were predicted to lose < 5% of their hydraulic capacity as opposed to 13% for the trees in the younger sites. Our results suggest that the responses to water stress of tree species in different forest ages greatly vary with a tendency of trees in younger sites to be more resilience than those in older sites. Such information would benefit the selection of tree species that could adapt well to specific environments, thus improving the strategies for managing forests of different ages under a warmer future.

## Introduction

Native tropical forests in Southeast Asia have been substantially converted to other forms of land use. In the past few decades, the rate of forest clearance in this region has been ranked among the highest in the tropics, with an average net loss of 1.6 million ha year^−1^ between 1990 and 2010^1^. Such land conversion is mostly attributed to many human activities, including commercial logging^2^, intense cultivation^3^, and food production^4^. The remaining degraded areas are then usually abandoned after several years of operation and transformed into secondary forests, whether by natural succession or human plantation. Consequently, forested areas in Southeast Asia consist of various stages of forest succession including primary forests and different phases of secondary forests. Evidence showed that tropical forests have been severely affected by extreme events from human-induced climate change, such as droughts, warmer temperature including heat waves, and fires^5,6^. Among these adverse impacts, droughts may have the greatest effect on forested areas worldwide^7^. In particular, the ongoing impacts from increases in frequency, duration and intensity of droughts are threatening the productivity and survival of forests^8^. Thus, droughts have been identified as a major contributing factor affecting forest physiological responses in many regions^5,9^, potentially accelerating the rates of tree decline and forest mortality^10,11^. Therefore, the combination of the impacts from anthropogenic disturbances and severe droughts will certainly be exacerbated in Southeast Asian forests, hence the need to improve the understanding of underlying mechanisms governing ecosystem functions in these mosaic patches of forests.

Forest structure and species composition in different forest stages differ^12^. Disparate vegetation structures, such as canopy height and density, differentiate microclimate and soil properties among successional stages^13^, thus affecting location, duration, and distribution of regenerated tree species within each stage^14^. Moreover, differences in canopy openness, tree density, vertical stratification, and the amount of plant litter cause variations in atmospheric temperature and humidity, and soil water availability along successional stages^15,16^. As a result, trees in secondary forests usually experience warmer and drier environments, compared to those in primary forests^17^. Since trees establishing in different succession may respond differently to droughts^18^, an interesting research question would be: how do trees in successional forests respond to water stress and droughts, which are predicted to be intensified under future climate? Studies that attempt to understand physiological mechanisms across forest succession have emerged in temperate and tropical ecosystems^19–22^, but rarely in Southeast Asia. This knowledge gap is a crucial limitation for the restoration of forests along successional gradients, as commonly observed in Southeast Asian forests, providing definitive recommendations for selecting tree species that are suitable for local environments.

Investigating responses of forests to changing environment relies on the understanding of drought-induced physiological mechanisms of dominant trees at the species level^23^. During drought conditions, species that can maintain appropriate hydration of cells and tissues by closing stomata potentially limit CO_2_ assimilation and tend to be susceptible to carbon starvation. In contrast, species that keep stomata open to maximize carbon uptake allow leaf water status to drop and therefore become vulnerable to xylem hydraulic dysfunction, also known as hydraulic failure. Indeed, different tree species would respond and adjust their functions in different ways to optimize carbon gain in relation to water loss^24,25^. Instead of carbon starvation, it has been revealed that hydraulic failure is most likely to be the cause of plant mortality triggering tree death from drought in tropical areas^26^. Since water is essential in many plant processes, its limitation could lead to many dysfunctions in terrestrial plants. One method to study the response of trees to drought-induced hydraulic failure is by quantifying their responses to water supply. Leaf water potential is a direct indicator of tree water status and represents the overall plant health^27^, providing a relative index of water stress that indicates how different tree species comparatively respond to changes in their surroundings^25,28^. Low water availability during droughts reduces soil, and stem and leaf water potentials, thus triggering cavitation-induced embolism in xylem conduits^27,29^, eventually leading to tree death^30^. Regarding such event, xylem vulnerability to embolism is often used to distinguish the drought tolerance of tree species^31^. It has been shown that the xylem vulnerability strongly relates to the ability of woody trees to survive and recover from periods of prolonged drought^32^. This hydraulic trait varies among species and is largely determined by differences in xylem structure^33^. Therefore, investigating the responses of tree species to drought-induced hydraulic failure and their xylem vulnerabilities will improve the knowledge of the limits of drought tolerance for woody tree species and determining trends in drought-induced forest mortality of different successional forests.

**Figure 1:**
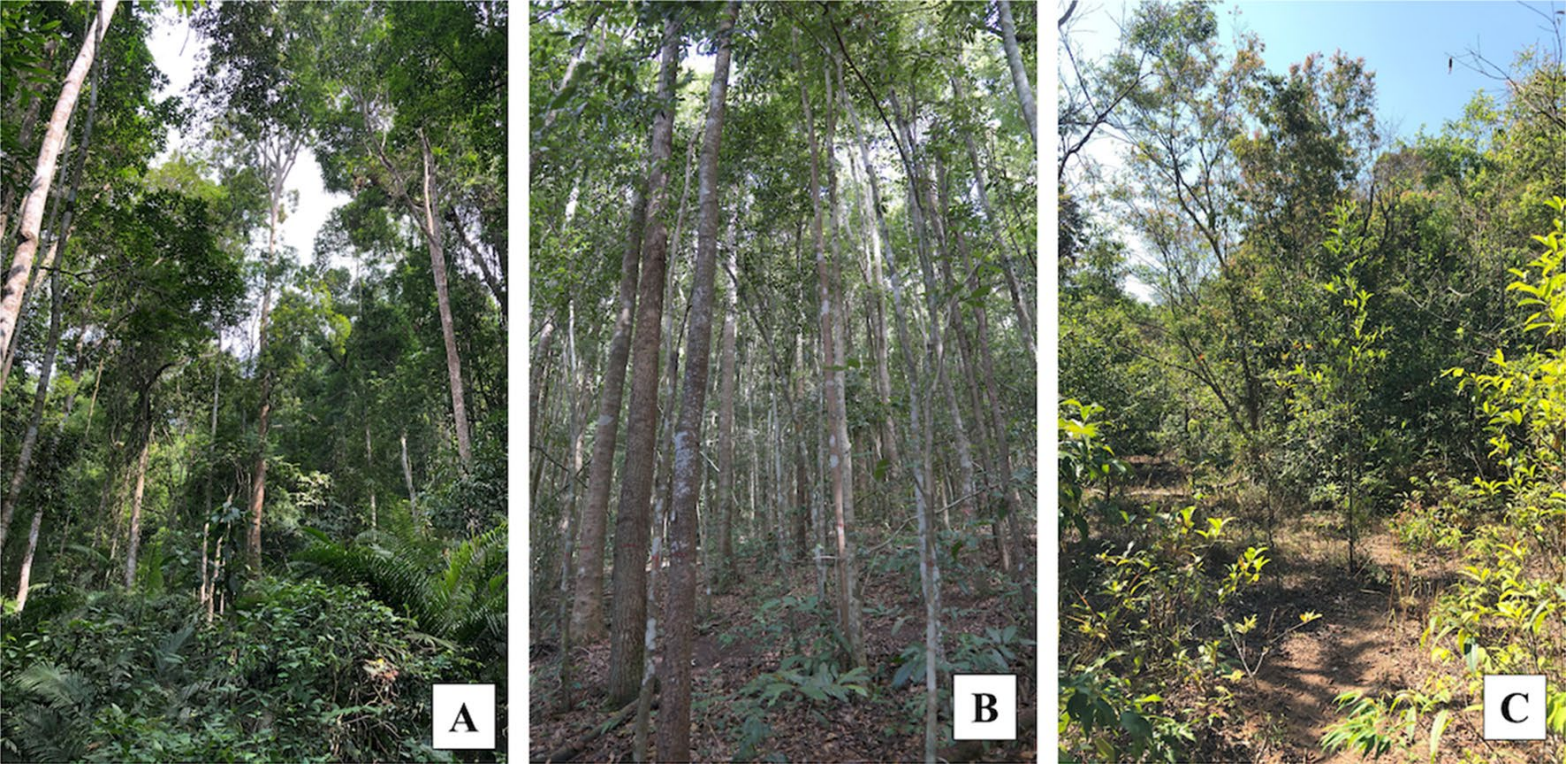
The studied sites in Khao Yai National Park for (**A**) old-growth forest (OF), (**B**) Intermediate forest (IF), and (**C**) Young forest (YF).

With these regards, this study aimed (1) to evaluate seasonal variation of leaf water status (water potential), and (2) to assess xylem vulnerability to embolism of dominant tree species from different forest successional stages located in Khao Yai National Park in Thailand, which is part of a UNESCO world heritage site. The study sites covered three forest stages: an old-growth forest (OF, > 200 years), an intermediate forest (IF, ~ 45 years), and a young forest (YF, ~ 5 years) as shown in Fig. 1. Particularly, we explored seasonal and site variations of leaf water status and drought tolerance in dominant tree species of these forests. The results revealed that the trees in the younger forests seemed to withstand changes in local environments than those in the old forest. The outcome from this study will fulfill the knowledge gap on species-specific hydraulic responses along the forest successional stages in tropical forests, which is needed for accurately modelling climate change impacts on forest health and vigor. Additionally, insights from this study can benefit policy decisions on tropical forest management, especially for selecting species to effectively restore forests in highly deforested and degraded regions, such as in Southeast Asia.

## Results

### Species selection and tree measurement

In this study, five dominant tree species in each forest stage were selected based on relative abundance of basal area, resulting in a total of 11 woody species with some species existing in multiple sites including *Schima wallichii* (OF and IF)*, Syzygium nervosum* (IF and YF), and *Symplocos cochinchinensis* (all sites). For each species, five individuals of similar stem diameter at breast height (10–20 cm DBH) were randomly selected for all measurements. Characteristics of the selected dominant tree species across the forest successional stages are summarized in Table 1.

**Table 1:** Characteristics of dominant tree species from the study sites of different forest succession in Khao Yai National Park. Relative basal area refers to the percentage of total basal area of the species to total basal area of all trees within the plot. Leaf habit shows whether the species is deciduous (D) or evergreen (E). For each species, diameter at breast height (DBH; cm), tree height (m) and sampling height (m), at which the sampled leaves were taken, are expressed in mean ± SD values of the sampled trees.

Tree species	Relative basal area (%)	Leaf habit	DBH (cm) of the sampled trees	Tree height (m)	Sampling height (m)
**Old-growth forest (OF)**					
*Dipterocarpus gracilis*	10.54	D	13.78 ± 3.27	17.16 ± 1.44	9.63 ± 3.00
*Sloanea sigun*	8.09	E	13.38 ± 2.19	13.52 ± 2.96	6.70 ± 0.98
*Ilex chevalieri*	5.00	E	16.74 ± 3.89	17.32 ± 1.58	6.90 ± 0.95
*Symplocos cochinchinensis*	3.40	E	16.00 ± 2.51	12.68 ± 3.54	9.77 ± 2.55
*Schima wallichii*	1.58	E	13.00 ± 1.98	12.04 ± 1.64	6.33 ± 1.04
**Intermediate forest (IF)**					
*Schima wallichii*	36.00	E	12.68 ± 1.90	10.60 ± 2.28	7.83 ± 2.31
*Machilus gamblei*	36.00	E	14.50 ± 3,76	21.14 ± 7.88	8.33 ± 1.29
*Eurya acuminate*	4.00	E	10.58 ± 0.76	9.52 ± 2.30	5.20 ± 0.75
*Symplocos cochinchinensis*	3.00	E	12.80 ± 2.09	11.82 ± 3.68	9.13 ± 2.22
*Syzygium nervosum*	2.00	E	13.34 ± 2.41	14.50 ± 5.82	8.20 ± 0.43
**Young forest (YF)**					
*Cratoxylum cochinchinense*	30.75	D	14.36 ± 2.67	9.30 ± 2.37	5.42 ± 0.13
*Syzygium antisepticum*	26.52	E	13.98 ± 1.70	9.34 ± 3.02	6.40 ± 1.25
*Adinandra integerrima*	12.08	E	15.28 ± 2.21	9.24 ± 2.63	4.61 ± 0.13
*Syzygium nervosum*	11.95	E	16.74 ± 2.97	8.80 ± 1.77	4.00 ± 1.73
*Symplocos cochinchinensis*	3.24	E	16.58 ± 3.26	9.98 ± 1.50	5.62 ± 1.20

### Seasonal variation in midday leaf water potential

**Figure 2:**
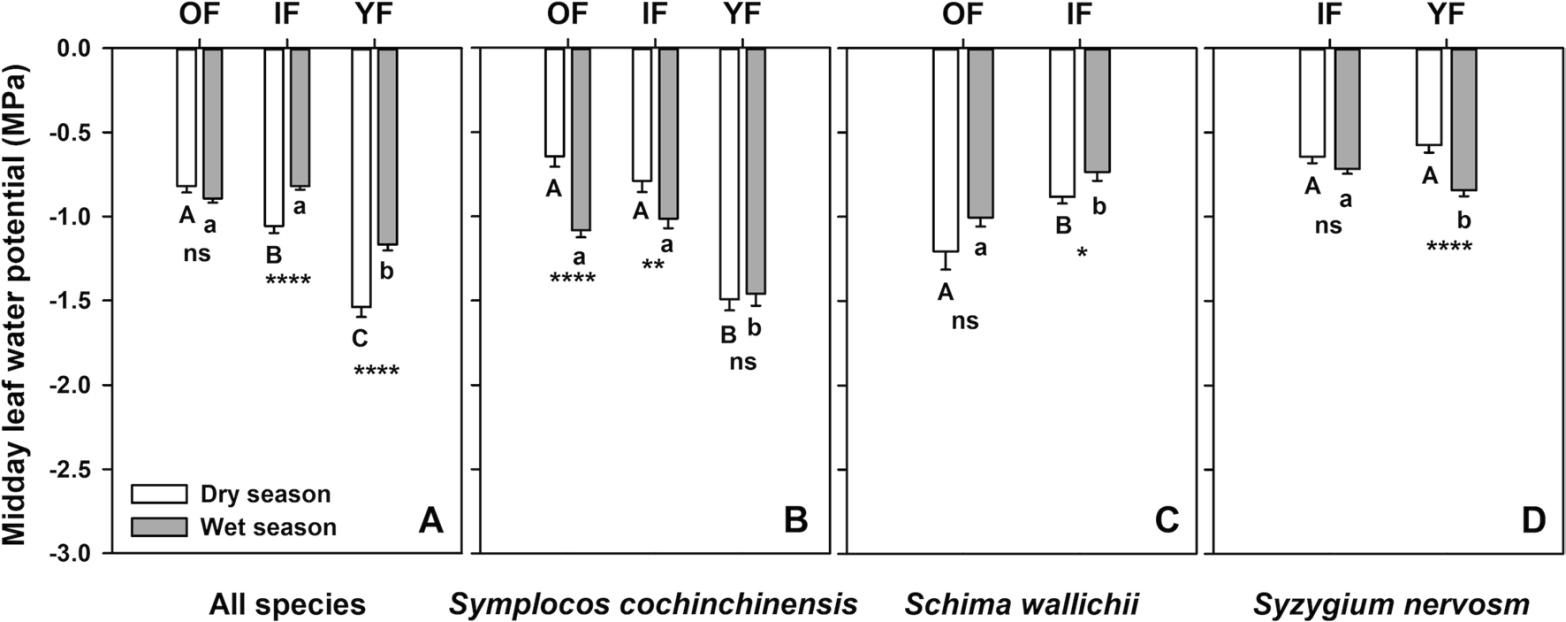
Midday leaf water potential (Ψ_md_) from the old-growth (OF), intermediate (IF) and young (YF) forests during the dry (white bars) and wet (gray bars) season in (**A**) all species and those growing in multiple successions including (**B**) *Symplocos cochinchinensis,* (**C**) *Schima wallichii* and (**D**) *Syzygium nervosum.* Each bar represents mean ± one standard error of 150 sampled leaves in (**A**), and 30 samples in (**B–D**). For each panel, different upper- and lower-case letters represent significant differences in the wet and dry seasons, respectively. Asterisks indicate significant seasonal differences within the same successional stage based on independent sample *t-test*.; ns = not significant, *p < 0.05, **p < 0.01, ***p < 0.001, and ****p < 0.0001.

Overall, seasonal variation in Ψ_md_ differed among the successional stages (Fig. 2A). Within each forest stage, IF and YF showed lower Ψ_md_ during the dry season than the wet season (*t*(221.00) = − 5.19, *p* < 0.0001 in IF and *t*(243.23) = − 5.45, *p* < 0.0001 in YF), with a slightly lower in YF than in IF (32% in YF vs. 29% in IF). However, there was no seasonal variation in Ψ_md_ found in OF (*t*(259.08) = 1.64, *p* = 0.10). Focusing on each season, the differences in Ψ_md_ were significant across forest successions in both seasons (*F*(2,447) = 61.71, *p* < 0.0001 in dry season and *F*(2,447) = 44.05, *p* < 0.0001 in wet season). It was more pronounced during the dry season, in which YF had the lowest Ψ_md_ (− 1.54 ± 0.059 MPa), followed by Ψ_md_ in IF (− 1.06 ± 0.041 MPa) and OF (− 0.82 ± 0.038 MPa), respectively. During the wet season, YF also had the lowest Ψ_md_ (− 1.17 ± 0.035 MPa) while Ψ_md_ in OF and IF were comparable (− 0.89 ± 0.025 MPa in OF and − 0.82 ± 0.021 MPa in IF).

Species growing in multiple sites exhibited different variations in Ψ_md_ between the seasons, and among the forest successions. For *S. cochinchinensis* (Fig. 2B), lower Ψ_md_ was observed in OF and IF during the wet season than in the dry season (*t*(57.58) = 5.21, *p* < 0.0001 in OF and *t*(47.61) = 2.86, *p* = 0.006 in IF), with a more negative of 69% and 28% in OF and IF, respectively. In contrast, no seasonal variation in Ψ_md_ of *S. cochinchinensis* growing in YF was found (*t*(57.69) = − 0.34, *p* = 0.73). For both seasons, Ψ_md_ differed significantly among the forest successions (*F*(2,87) = 48.34, *p* < 0.0001 in dry season and *F*(2,87) = 17.22, *p* < 0.0001 in wet season), in which the lowest Ψ_md_ was found in YF, while it was similar in OF and IF. For *S. wallichii*, seasonal difference in Ψ_md_ was detected only in IF (*t*(53.67) = − 2.25, *p* = 0.028), with a 19% lower Ψ_md_ during the dry season than in the wet season (Fig. 2C). For both seasons, lower Ψ_md_ was found in OF than in IF (*t*(36.50), *p* = 0.008 in dry season and *t*(57.99) = 3.63, *p* = 0.0006 in wet season). Only *S. nervosum* in YF differed between seasons (*t*(55.71) = 4.78, *p* < 0.0001), with a 47% lower in Ψ_md_ during the wet season than in the dry season (Fig. 2D). In the wet season, Ψ_md_ in YF was significantly lower than in IF (*t*(55.87) = 2.77, *p* = 0.007), whereas no difference between the forest successions was observed in the dry season (*t*(56.82) = − 1.19, *p* = 0.237).

### Vulnerability to xylem embolism

The species from three forest stages showed comparable mean xylem vulnerability to embolism (P_50_) (*F*(2,42) = 2.20, *p* = 0.123, Table 2) whereas great interspecific variation was observed in the same forest stage. P_50_ ranged from potentially more vulnerable in OF (− 2.87 ± 0.30 MPa) to less vulnerable in IF (− 3.04 ± 0.18 MPa) and in YF (− 3.71 ± 0.39 MPa). However, significant difference in sensitivity to xylem embolism (S) was observed among the successional forests (*F*(2,42) = 5.08, *p* = 0.011, Table 2). S was higher in OF (73.70 ± 21.60% MPa^−1^) than in IF (26.80 ± 1.25% MPa^−1^) and YF (23.40 ± 1.05% MPa^−1^). At the forest level, the percentage loss of hydraulic conductivity corresponding to Ψ_md_ during the dry season (PLC_dry_) did not show a clear pattern along the forest successions. OF tended to have similar PLC_dry_ to IF (4.61 ± 0.97% in OF and 12.90 ± 2.14% in IF); however, PLC_dry_ in IF also appeared to be comparable to that in YF (18.40 ± 4.93% in YF). For the species occupying multiple forest stages, vulnerability to xylem embolism was generally comparable across forest successions. *S. cochinchinensis* exhibited similar P_50_ across successions (*F*(2,6) = 0.40, *p* = 0.687), by having P_50_ of − 3.27 ± 0.04 MPa in OF, − 3.39 ± 0.03 MPa in IF, and − 3.24 ± 0.21 MPa in YF. The same trend was also found in *S. nervosum* (*t*(2.12) = − 0.34, *p* = 0.762), by having P_50_ of − 2.30 ± 0.17 and − 2.24 ± 0.03 MPa in IF and YF, respectively. In contrast, significant difference in P_50_ was found in *S. wallichii* between the two forest successions (*t*(3.22) = 4.63, *p* = 0.016), with 0.45 MPa lower in OF than in IF.

**Table 2:** Vulnerability to xylem embolism, represented by xylem pressure at 50% loss of hydraulic conductivity (P_50_), slope of the vulnerability curve (S), and percentage loss of hydraulic conductivity corresponding to midday leaf water potential during the dry season (PLC_dry_) of selected dominant tree species from different successions in Khao Yai National Park, Thailand. For a given species, values are means ± one standard error of 3 sampled branches that we used to generate the vulnerability curve separately. For each successional stage, values presented are averaged from the selected dominant tree species with one standard error (n = 15). Different upper- and lower-case letters represent significant differences among the forest successions and within each forest succession, respectively (One-way ANOVA with Tukey’s post-hoc test, significance level of 0.05).

Tree species	P_50_ (MPa)	S (% MPa^−1^)	PLCdry (%)
**Old-growth forest (OF)**	− **2.87 ± 0.30 A**	**73.70 ± 21.60 A**	**4.61 ± 0.97 A**
*Dipterocarpus gracilis*	− 0.89 ± 0.01 d	222.56 ± 45.72 b	3.06 ± 2.22 a
*Sloanea sigun*	− 2.55 ± 0.21 c	43.83 ± 10.92 a	7.10 ± 3.32 a
*Ilex chevalieri*	− 4.17 ± 0.08 a	41.09 ± 11.49 a	2.00 ± 1.36 a
*Symplocos cochinchinensis*	− 3.27 ± 0.04 b	33.34 ± 2.73 a	3.21 ± 0.94 a
*Schima wallichii*	− 3.46 ± 0.08 b	27.81 ± 2.17 a	7.69 ± 0.85 a
**Intermediate forest (IF)**	− **3.04 ± 0.18 A**	**26.80 ± 1.25 B**	**12.90 ± 2.14 AB**
*Schima wallichii*	− 3.01 ± 0.05 bc	23.58 ± 0.17 a	11.85 ± 0.37 ab
*Machilus gamblei*	− 2.46 ± 0.34 c	24.36 ± 1.96 a	25.51 ± 3.94 c
*Eurya acuminate*	− 4.01 ± 0.10 a	24.44 ± 1.59 a	9.57 ± 1.90 ab
*Symplocos cochinchinensis*	− 3.39 ± 0.03 ab	34.35 ± 2.45 b	2.88 ± 0.69 a
*Syzygium nervosum*	− 2.30 ± 0.17 c	27.22 ± 1.61 ab	14.50 ± 1.94 b
**Young forest (YF)**	− **3.71 ± 0.39 A**	**23.40 ± 1.05 B**	**18.40 ± 4.93 B**
*Cratoxylum cochinchinense*	− 2.39 ± 0.24 d	20.73 ± 2.63 a	52.89 ± 4.51 d
*Syzygium antisepticum*	− 4.71 ± 0.08 b	20.74 ± 0.44 a	6.51 ± 0.72 ab
*Adinandra integerrima*	− 5.97 ± 0.06 a	24.44 ± 1.26 ab	1.43 ± 0.24 a
*Syzygium nervosum*	− 2.24 ± 0.03 d	29.05 ± 1.64 b	12.68 ± 0.92 bc
*Symplocos cochinchinensis*	− 3.24 ± 0.21 c	21.87 ± 1.54 ab	18.25 ± 1.54 c

**Figure 3:**
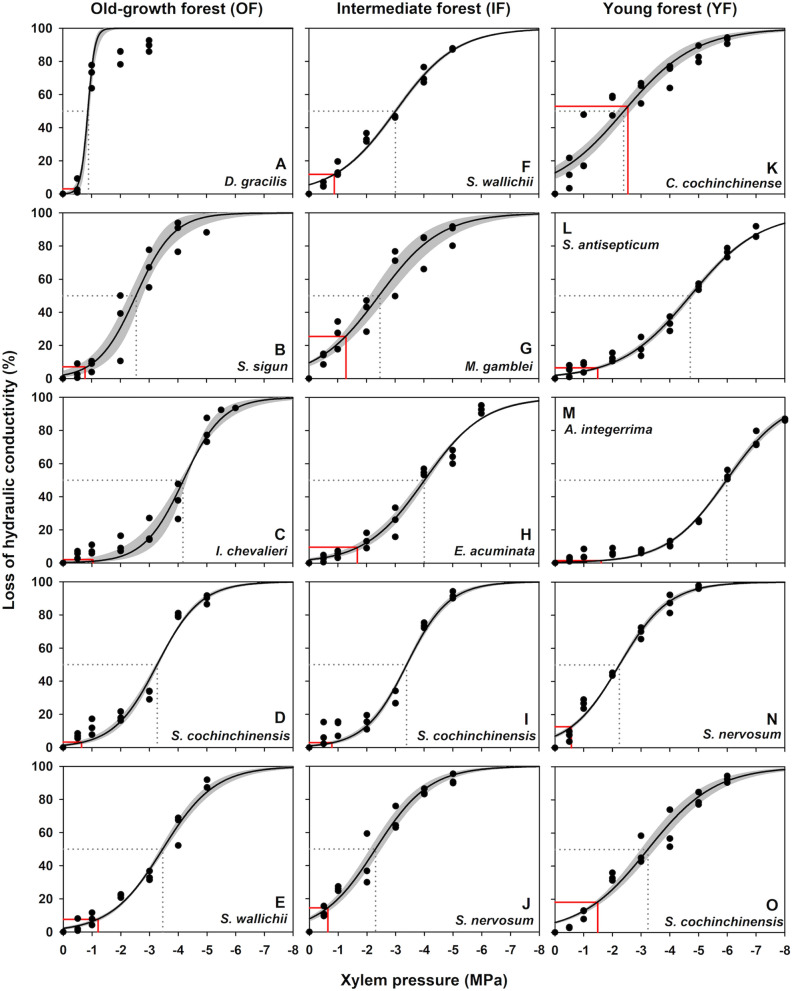
Vulnerability of xylem to embolism of branches from dominant tree species from different successions in Khao Yai National Park, Thailand. Mean vulnerability curves are presented with shaded bands representing one standard error from 3 measured branches for a given species. The gray dotted and solid red lines indicate the xylem pressure at 50% loss of hydraulic conductivity (P_50_) and percentage loss of hydraulic conductivity corresponding to midday leaf water potential (Ψ_md_) during the dry season (PLC_dry_), respectively.

Within each forest successional stage, xylem vulnerability varied greatly among the dominant tree species (Table 2, Fig. 3). In OF, the lowest P_50_ was found in *I. chevalieri* (− 4.17 ± 0.08 MPa, Fig. 3C) whereas the highest P_50_ was found in *D. gracilis* (− 0.89 ± 0.01 MPa, Fig. 3A). Slopes of vulnerability curves were comparable across the dominant tree species in OF, except for *D. gracilis* that had the steepest slope than the others. PLC_dry_ did not differ substantially across species in OF. In IF, *E. acuminata* (Fig. 3H) was the most while *S. nervosum* (Fig. 3J) was the least resistant to xylem embolism (− 4.01 ± 0.10 MPa and − 2.30 ± 0.17 MPa, respectively) compared to other coexisting species. Sensitivity to xylem embolism was higher in *S. cochinchinensis* than the rest of the species in IF. *S. cochinchinensis* in IF also responded less to potentially dry conditions in the dry season by losing relatively small hydraulic conductivity at midday (2.88 ± 0.69%, Fig. 3I) while *M. gamblei* in IF reacted more by further losing the hydraulic conductivity in the dry season (25.51 ± 3.94%, Fig. 3G). In YF, *A. integerrima* (Fig. 3M) displayed the lowest P_50_ value at − 5.97 ± 0.06 MPa, with low response to dry conditions as observed from midday PLC_dry_ (1.43 ± 0.24%). *C. cochinchinense* (Fig. 3K), however, had the highest P_50_ (− 2.39 ± 0.25 MPa), with the greatest response from losing hydraulic conductivity in the dry season (52.89 ± 4.51%) compared to the other species. The steepest slope of vulnerability curve in YF was measured in *S. nervosum.*

## Discussion

Midday leaf water potentials (Ψ_md_) and xylem vulnerability (P_50_) are summarized from previous studies in tropical forests across the globe (Table S1). Due to differences in experimental conditions and settings, these studies could not be directly compared to the results from this study. Nonetheless, measured values of Ψ_md_ from this study were within the ranges of those observed in Neotropics^21,26,34,35^, Australasia^36,37^, and Indomalaya^38–40^. For xylem vulnerability, results from this study were within similar ranges of those from Neotropics^26,34^ and Indomalaya^38,40^, but were lower than those shown in the other studies in Table S1. In general, this comparison reveals that leaf water potential and xylem vulnerability vary greatly within tropical region, and they seem to be site-specific.

The dominant tree species across forest successions showed different seasonal variations in Ψ_md_. At forest level, YF exhibited lower Ψ_md_ in both wet and dry season and had greater seasonal variation in Ψ_md_ compared to OF and IF (Fig. 2A). This could imply that lower Ψ_md_ during water-limited conditions, e.g., in the dry season, and higher variation in Ψ_md_ induced by seasonal changes could occur frequently in the younger successions, consistent with other studies^34,41,42^. In contrast to late successional forests, early successions receive more direct solar heating owing to more open canopy, leading to drier conditions in the atmosphere and higher temperature^12^. A drier environment in early successional stage would introduce greater water stress than in late succession and become more intensified during the onset of dry season^18^, as seen by lower Ψ_md_ in drier YF site as compared to moister OF and IF sites. Due to certain limitations during the COVID-19 pandemic, we could not conduct the measurement to cover the entire dry season. However, we hypothesize that if we had continued collecting data throughout the dry season, leaf water status would have become lower in all species and sites as soil would have become even drier. Furthermore, for each successional stage, more distinct differences in seasonal variation in Ψ_md_ could be expected from each dominant tree species, especially in drier sites. Interestingly, studies have shown that species found in drier sites have higher tolerance to desiccation, meaning the ability to remain active at lower water potentials^43–45^. However, allowing low Ψ_md_ in drier sites can also be detrimental for dehydration and hydraulic failure, especially during the dry season, if the value decreases beyond the critical threshold of xylem embolism^46^.

To further investigate the variations in Ψ_md_ by excluding potential confounding effects from various intra-site species, species existing in multiple sites were examined. As would be expected from its existence in drier environment, *S. cochinchinensis* in YF exhibited the lowest Ψ_md_ in both seasons compared to OF and IF, with no significant seasonal difference in Ψ_md_ (Fig. 2B). *S. cochinchinensis* in OF and IF showed comparable Ψ_md_ in both seasons; however, their Ψ_md_ during the wet season was lower than in the dry season, despite the presumably better access to water in the wet season. This unexpected pattern also occurred in *S. nervosum* in YF, when lower Ψ_md_ was found in the wet season compared to the dry season (Fig. 2D). Such unexpected patterns may be explained by that, during the wet season, *S. cochinchinensis* in OF and IF and *S. nervosum* in YF adopted a less conservative water use regulation when water is more abundant. This observation was also reported in some previous studies^47,48^, with no clear explanation being provided. In *S. wallichii*, lower Ψ_md_ was found in OF than in IF in both seasons (Fig. 2C), with no seasonal difference in OF but a slight change in IF in the dry season. Even though they existed in a relatively wetter environment, the sampled trees of *S. wallichii* in OF grew under a large gap created by tree falls. The higher intensity of light under the gap strongly influence the microclimate, leading to higher air temperature and lower air humidity compared to the adjacent area^49,50^. Therefore, the leaf-level water deficits resulted from canopy gap may contribute to the unexpectedly more negative values of Ψ_md_ in OF than in IF. Different changes and patterns in Ψ_md_ induced by seasonal changes found in this study seem to be species-specific. These different strategies dealing with water-limited condition have a critical impact on xylem embolism^51,52^, and hence the potential risk of tree mortality resulted from water stress-induced hydraulic failure.

Many studies have documented that vulnerability to xylem embolism vary substantially among trees from different habitats. Species occupying in drier sites are generally less vulnerable to embolism than those occupying in wetter sites^34,38,53–55^. Our results revealed a tendency for species at more xeric sites to have higher ability to resist xylem embolism, despite non-statistical difference at the forest level (Table 2, Fig. 3). The most vulnerable species to xylem embolism was found in OF, a deciduous tree species *D. gracilis* with the highest P_50_ of − 0.89 MPa. On the other hand, *A. integerrima*, an evergreen tree species in YF, had the lowest P_50_, at − 5.97 MPa, standing out from the rest of the studied tree species from all forest successions. The presence of species with lower P_50_ in drier sites could imply the adaptive importance of embolism resistance in response to the environments where water stress is more pronounced^33^. In addition, the overall sensitivity to xylem cavitation in OF was considerably higher than that in IF and YF, potentially suggesting the higher rate of embolism occurrence for the species in the wetter site. Choat, et al.^53^ showed that populations of a common species, *Cordia alliodora* in Costa Rican tropical forests were less resistant to embolism in the wetter sites than those growing in the drier sites, suggesting their adjustment in hydraulic traits to establish themselves in a wide range of habitats. In this study, however, species occupying in multiple forest stages, e.g., *S. cochinchinensis*, exhibited comparable P_50_, implying the similar embolism resistance across the forest successions. The lack of difference in P_50_ along forest successions was also observed in a tropical dry forest in Mexico^17^. Great variations in the xylem vulnerability among species imply that evaluating species’ performance under water stress should be carefully interpreted, as other mechanisms, e.g., stomatal regulation, sapwood water storage, or leaf-shedding strategy, could also contribute to xylem resistance to embolism^56–59^.

In each forest succession, larger variations in embolism resistance were found in OF and YF than in IF, based on their coefficients of variation (40.42%, 22.89%, and 40.43% in OF, IF, and YF, respectively). Cartwright et al.^60^ suggested that substantial variability in drought response within an ecosystem can be driven by endogenous factors (e.g., phenological characters) and by exogenous factors (e.g., topographic and hydrologic characteristics). In this study, differences in leaf phenology among the dominant tree species were observed in OF and YF. Compared to the other evergreen species within the same site, deciduous tree species, *D. gracilis* in OF and *C. cochinchinense* in YF, exhibited lower resistance to xylem embolism. Such difference in xylem vulnerability between deciduous and evergreen tree species was also found in the studies from other tropical forests^39,59,61^ and subtropical forests^62^. In addition, other exogenous factors may contribute to the variation in P_50_, particularly in OF. The variation in xylem vulnerability in OF could be explained by high microhabitat heterogeneity in this area^63^, that might lead to spatial distribution of vegetation with varying sensitivity to water availability within the site. For example, species with presumably better access to water, e.g., *S. sigun* which dominated in flat lowland near streamside, showed higher vulnerability to xylem embolism among the others. In contrast, species with limiting soil water, e.g., *I. chevalieri* which occupied in a hilly slope, showed relatively less vulnerable to embolism compared to the rest of the species. Consistent with this finding, Zhang et al.^64^ and Zhu et al.^38^ found a wide range of P_50_ in tropical karst forests, in which species existing in the middle to top of hilly areas were more resistant to embolism than species dominated in lowlands or valleys, resulting from soil water gradient. Nevertheless, further investigations on hydraulic architecture, sapwood water storage capacity, and rooting depth should be performed to confirm such findings.

In terrestrial plants, the ability to sustain xylem water transport under water deficit conditions is crucial for plant functions and survival. Our results showed that the dominant tree species from each succession experienced midday leaf water potentials during the dry season that could result in loss of hydraulic conductivity (PLC_dry_) at different levels (Table 2, Fig. 3). Based on xylem vulnerability curves and Ψ_md_, most of the studied tree species operated well below or close to their P_50_ values. PLC_dry_ tended to be lower towards the older sites, which was similar to a study showing small increase in xylem embolism during the dry season in an old and natural forest in Thailand^65^. Moreover, species with higher resistance to xylem embolism tended to lose lower hydraulic conductivity during the dry season across the successions. For example, species with high embolism resistance, e.g., *I. chevalieri* in OF, *S. cochinchinensis* in IF, and *A. integerrima* in YF, showed lower than 3% in PLC_dry_. The reverse was seen in species that were more vulnerable to embolism, e.g., *S. sigun* in OF, *M. gamblei* in IF, *C. cochinchinense* in YF, which exhibited 7% to 53% in PLC_dry_. The probability of losing higher xylem water transport efficiency related to the tension experienced during the dry season may be associated with species’ performance to deal with embolism, consistent with findings obtained from a karstic woodland^66^, an Amazonian tropical forest^59^, and across forest biomes^67^. This result, thus, implies the significance of embolism resistance in determining species risk of hydraulic dysfunction during low water availability^34,68,69^.

Understanding how plants respond to water stress from different tree species and different forest successions is useful not only for the forest conservation and restoration efforts, but also for the predictions of tree mortality across the successions. In accordance with predicted warming atmosphere and more variable droughts, it is important for forest restoration and conservation to consider whether or not young seedlings can establish^70^, as well as the threshold at which tree mortality would occur^71^. This could provide necessary information in order to maintain and promote the species that could adapt well in particular environments, under both current and future conditions. By selecting species that could be well-adapted in a specific setting, e.g., using information derived from P_50_ and PLC_dry_, the likelihood of success of forest restoration and conservation in a drier and hotter future could be enhanced^72–74^.

## Methods

### Study site

The study site was in Khao Yai National Park, Thailand (14º26´31ʺ N, 101º22´55ʺ E). The average elevation ranges 700–800 m above sea level. This region is dominated by monsoon climate, where the dry season usually lasts from November to April and from May to October for the wet season^63^. Based on data (1994–2014) of a weather station, ~ 3 km away from the study sites, the mean annual temperature was 22.4 °C, with monthly temperature ranging from 19.4 °C in December to 24.3 °C in April. The mean annual rainfall was 2,073 mm. Permission to study in these sites were granted by the Department of National Parks, Wildlife, and Plant Conservation of Thailand.

Khao Yai National Park comprises the mosaic fragments in different forest successional stages, such as old-growth forests and different aged secondary forests. These secondary forests naturally regenerated from either natural disturbance (i.e., fires or fallen trees) or anthropogenic impacts (i.e., deforestation or land conversion). In this study, three forest stands representing different forest successional stages including an old-growth forest (OF), an intermediate forest (IF), and a young forest (YF) were selected. The OF (Fig. 1A) was in the 30-ha Mo Singto forest dynamics plot, one of the ForestGEO plots of the Centre for Tropical Forest Science (CTFS) network^75^, with the age > 200 years^63^. This forest has several tree layers, with the mean canopy height of 45 m, a leaf area index (LAI) of 5, and stem density of 1,112 trees ha^−1^^76^. The IF (Fig. 1B) was located in the northern side of OF. This 45-year-old plot had an area of 1 ha, with an average canopy height of 25 m, a LAI of 6, and stem density of 2,052 trees ha^−1^^77^. The YF (Fig. 1C) was located approximately 3 km away in the southeastern direction of OF. This 5 year-old forest had an area of 2 ha with a stem density of 1,226 trees ha^−1^^77^. The YF’s mean canopy height was 15 m, with sparser vegetation coverage compared to the other successional forests. The soil type and soil texture in these forests were classified as gray-brown ultisol and as sandy clay-loam to clay loam, respectively^78^.

### Species selection

The dominant tree species in each forest stage were chosen based on their relative abundance, which was calculated from the basal area of one species relative to total basal area of all species within the site. Then, five dominant tree species and five individuals per species from each of the three successional forests were selected for all measurements, resulting in a total of 75 trees sampled and 11 different species, with some species existing in multiple sites (Table 1), including *Schima wallichii* (OF and IF)*, Syzygium nervosum* (IF and YF), and *Symplocos cochinchinensis* (all sites). A summary of characteristics of dominant tree species is shown in Table 1, and detailed information about the flora and characteristics of selected species is described in Brockelman, et al.^63^. According to Brockelman, et al.^63^, *Dipterocarpus gracilis* and *Cratoxylum cochinchinense* are deciduous by shedding their leaves and stipules during February–March but are never completely leafless (field observations during the measurements). The other sampled species are evergreen. A large canopy gap resulted from fallen trees was also observed around the sampled trees of *S. wallichii*, and some of the sampled trees of *S. cochinchinensis*. The sampled trees in IF existed in a hilly area, where its canopy coverage was more homogeneous and denser compared to that of OF and YF^76^. Overall, all sampled trees in YF experienced drier conditions and stronger radiation from more open canopy in contrast to OF and IF^76,79^.

### Measurement of midday leaf water potential

**Figure 4:**
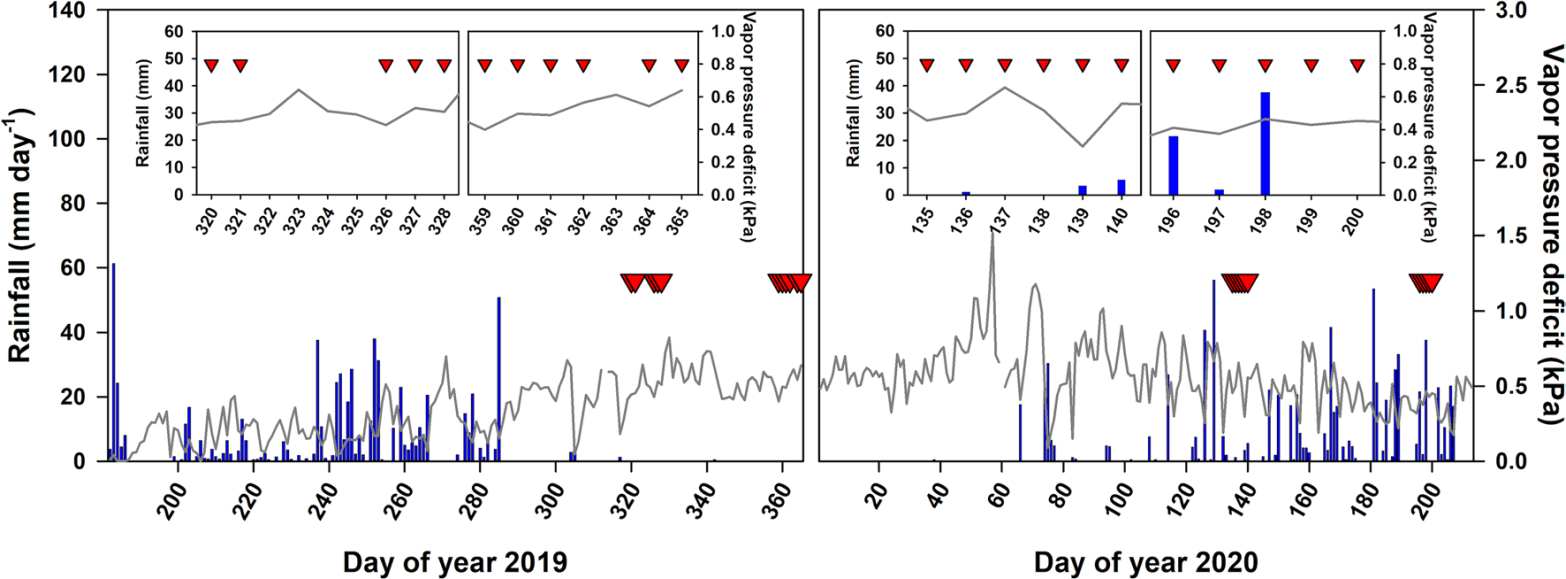
Distribution in rainfall (blue bars) and vapor pressure deficit (VPD, gray line) from 1st July 2019 (day of year 182 in 2019) to 30th July 2020 (day of year 213 in 2020) at Khao Yai National Park, Thailand. Red triangles indicate the sampling days for midday leaf water potential (Ψ_md_) measurements. Inset figures are the zoomed-in version of daily rainfall and VPD patterns during the Ψ_md_ sampling periods.

Midday leaf water potential (Ψ_md_) was measured with a Scholander pressure chamber (Model 1505D-EXP, PMS instrument, Albany, OR, USA) on samples taken between 10:00 and 14:00 h in all study sites. The measurement was conducted twice during each of the dry (November to December of 2019) and the wet season (May to July of 2020) to characterize seasonal variation in Ψ_md_. For each tree species, five individuals with similar stem diameter (10–20 cm DBH, Table 1) were chosen. For each individual tree, three healthy leaves fully exposed to sunlight were randomly selected from the bottom and outermost branches^80^. Each leaf was cut with a razor blade and placed inside the pressure chamber with its cut end of the leaf stalk protruding from the sealing port. The chamber was then gradually pressurized using nitrogen gas (N_2_) until a drop of water appeared at the cut surface of the stalk. The balancing pressure inside the chamber, which is equivalent to Ψ_md_, was then recorded. To avoid the potential loss of water from the leaves, this measurement was conducted immediately after the leaves were collected. Meteorological data concomitant to the measurements of Ψ_md_ (Fig. 4, inset figures) were obtained from the weather station located at Khao Yai National Park headquarter, which was about 3 km away from the forest plots. During the measurement periods, rainfall was not observed, except in the wet season during which rainfall occurred mostly at night. The average daily vapor pressure deficit (VPD) from the sampling days during the dry and wet seasons were similar (independent sample *t-test*, *t*(18.22) = 0.983, *p* = 0.338), averaging 0.50 ± 0.07 kPa and 0.46 ± 0.09 kPa, respectively. In addition, soil moisture was measured using a soil moisture probe (SM150T, DeltaT Devices, London, UK) around the sampled trees in each study site during the onset of the dry season (February 2020), which did not coincide with our measurement campaigns, and compared across the three sites. The differences in soil moisture among forest stages were significant (one-way ANOVA, *F*(2,222) = 174.92, *p* < 0.0001), in which OF had the highest mean soil moisture (45.4 ± 8.7%), followed by IF (37.8 ± 7.0%), and YF (23.9 ± 5.3%), respectively.

### Measurement of xylem vulnerability to embolism

Before performing the measurement, branch maximum xylem vessel lengths (MVL) were estimated for each species to assess the minimum sample size useable to avoid the introduction of air artifacts due to open xylem elements^81,82^. By using the air infiltration technique^83–85^, the estimation of MVL was made in the study sites on three branches collected from the same individuals used for Ψ_md_ measurement. Branches ranging between 0.6–1.0 m in length and 5–10 mm in diameter were cut, connected to a rubber tubing with a syringe at the basal end, and immersed the other end in water. Then, a small air pressure from a syringe was applied to the basal end, while the other end was shortened at 1 cm intervals until the air bubbles emerged. The MVL was determined from the remaining length plus 1–2 cm. Overall, the MVL of branches from all the selected tree species was within the range of 15–50 cm (Table S2). Thus, for each species, three straight branches with similar diameter (5–10 mm) and length around 50 cm were collected and kept immersing in cold water (~ 4 ºC) before transporting to the laboratory for the measurement of xylem embolism.

Xylem vulnerability to water stress-induced embolism was measured using air-injection technique^86^ and xylem specific conductivity (K_s_) was determined following Melcher, et al.^87^. The air pressure technique gave reliable results because most of the studied species were diffuse-porous species, and the length of the samples were at least four times the length of the pressure sleeve^82^. First, the collected branch segments were flushed at pressure of 100 kPa for 25–30 min to remove air emboli with perfusing solution, de-ionized and ultra-filtered water (PURELAB Chorus 1 Complete, ELGA LabWater, Woodridge, IL, USA) that was degassed and adjusted to an acidic pH (2–3) with HCl. This removal allowed the segments to restore their maximum conductivity (K_smax_). Each segment was then connected to a tubing apparatus with the basal end attached to the perfusing solution reservoir (upstream) and the other end connected to a pipette (downstream). Next, the flow rate through the segment was measured and K_smax_ was calculated according to Darcy’s Law^88^:

1 $${\text{K}}_{\text{s}} = \frac{\text{Ql}}{\Delta{\text{P}{\text{A}}_{\text{s}}}}\frac{\upeta}{{\upeta}_{0}}$$

where K_s_ is the xylem specific conductivity in kg m^−1^ s^−1^ MPa^−1^, Q is the flow rate of fluid (kg s^−1^), l is the length of the segment (m), ∆P is the pressure difference between two ends of the segment (MPa), As is the sapwood cross-sectional area (m^2^), η is the viscosity of the fluid at the temperature when the experiment is performed (N s m^−2^), and η_0_ is the reference viscosity at 25 °C (N s m^−2^). After determining K_smax_, the segment was placed inside a double-ended pressure sleeve (PMS Instrument Company, Albany, OR, USA). The chamber was then connected to a pressure chamber (same instrument used for Ψ_md_ measurements) and pressurized with N_2_ to artificially induce embolism^89^. First, the chamber was applied with a small pressure, 0.5 MPa, and maintained for at least two minutes before reducing the pressure back to atmospheric level. After the pressurization, the segment was rested for 10–30 min for the balanced system and K_s_ with induced embolism was determined. This procedure was repeated by increasing the injection pressure from 0.5 or 1 MPa steps (depending on species), until more than 85% loss of K_s_ was reached. The percentage loss of hydraulic conductivity (PLC) was calculated as:

2 $$\text{PLC} = 100 \times \left(1 - \frac{{\text{K}}_{\text{s}}}{{\text{K}}_{\text{smax}}}\right)$$

where K_s_ is the xylem specific conductivity following each step of increased pressure and K_smax_ is the maximum conductivity measured after removal of embolism. Then, xylem vulnerability curves were created by plotting PLC as a function of the applied pressures and fitted by the following sigmoidal equation described by Pammenter and Vander Willigen^90^ and modified by Domec and Gartner^91^:

3 $$\text{PLC} = \frac{100}{(1 + \text{exp}(\text{S}/25\text{(P} - \mathrm{P}50{)))}}$$

where P (MPa) is the applied pressure, and S (%PLC MPa^−1^) is the slope of linear part of the of the vulnerability curve and is centered on P_50_ (MPa), which is the pressure causing 50% loss of xylem conductivity and commonly used to compare embolism resistance among and between species. The parameter S represents the sensitivity of a species to xylem pressure-induced embolism^37^. Additionally, the constructed vulnerability curves were also used to calculate the percentage loss of hydraulic conductivity corresponding to midday leaf water potential during the dry season (PLC_dry_) to further assess the potential water transport efficiency during water-limited conditions from each studied tree species.

### Data analysis

To accomplish the first objective, two sets of analysis were performed to detect and confirm significant differences in Ψ_md_ across successional stages and seasons. The first set was to test for overall difference among successional stages using combined data from all dominant species within each succession, while the second set was to further evaluate the difference among forest stages by focusing only on the data from species found in multiple sites. For the first set of the analysis, one-way analysis of variance (ANOVA) was performed to evaluate the significant difference of Ψ_md_ among forest successions in the same season. Then, in each succession, an independent sample *t-test* was used to test the significant difference in Ψ_md_ between the wet and dry seasons. For the second set of the analysis, Ψ_md_ from the species occupying in all study sites, i.e., *Symplocos cochinchinensis*, was tested for the significant difference across the forest succession in each season by using one-way ANOVA and tested the significant difference in seasonal variation by using independent sample *t-test*. For the species occupying in two sites, i.e., *Schima wallichii* in OF and IF and *Syzygium nervosum* in IF and YF, significant differences in Ψ_md_ between the forest succession and seasons were evaluated using independent sample *t-test*. Tukey’s post hoc test was used after the one-way ANOVA test to determine which pairwise comparisons are significantly different. To characterize differences in xylem vulnerability to embolism between species and sites (objective 2), regression analysis using sigmoidal equation (Eq. 3) was conducted to generate P_50_, S, PLC_dry_ for all selected tree species. P_50_, S, and PLC_dry_ were also tested using one-way ANOVA with Tukey’s post-hoc test to evaluate the significant differences among forest successions by using pooled data from the dominant tree species, and across the dominant tree species within each forest succession. All analyses in this study were performed using R (version 4.0.3) and all statistical tests were considered at the significance level of 0.05. All graphs and regression analysis were made by SigmaPlot 12.0 (Systat Software, Inc., San Jose, California, USA).

## Supplementary Information

Supplementary Information.

## Data Availability

The datasets used and/or analyzed during the current study available from the corresponding author on reasonable request.
