# Supplementary material for: Variations in leaf water status and drought tolerance of dominant tree species growing in multi-aged tropical forests in Thailand
Source: Sci Rep. 2022 Apr 27;12:6882. doi: 10.1038/s41598-022-10988-1 (PMC9044374; doi:10.1038/s41598-022-10988-1)
Supplement: Supplementary file 1 — Supplementary Information. [file 41598_2022_10988_MOESM1_ESM.docx]

**Supporting information**

**Article title**: Variations in leaf water status and drought tolerance of dominant tree species among three successional forests in Southeast Asia

**Authors**: Weerapong Unawong, Siriphong Yaemphum, Anuttara Nathalang, Yajun Chen, Jean-Christophe Domec, and Pantana Tor-ngern

Table S1 Literature survey of leaf water potential (Ψ, MPa) and xylem tension at 50% loss of hydraulic conductivity in branches or stems (P_50_, MPa) in tropical forests including this study. Values are shown as ranges. NA indicates not available data.

| Location | Condition | Dominant tree species | Ψ  (MPa) | | P_50_ (MPa) | Reference |
| --- | --- | --- | --- | --- | --- | --- |
| Tropical rain forest in Caxiuana National Forest, State of Pará, Brazil  (1°43′S, 51°27′W) | Control | *Eschweilera* sp., *Licania* sp., *Pouteria* sp., *Protium* sp., *Swartzia* sp., and *Inga* sp. | NA | | -0.80 to  -4.50 | Rowland et al. (2015) |
|  | Through-fall exclusion (TFE) |  | NA | | -0.87 to  -4.20 |  |
| Tropical rain forest in Caxiuana National Forests, State of Pará, Brazil  (1°44’13.2”S, 51°27’28.8”W) | Midday Ψ was measured in control (left in Ψ column) and TFE (right in Ψ column) experiments.  P_50_ was estimated during throughfall exclusion experiment (TFE). | *Inga* sp., *Eschweilera* sp., *Protium* sp., and *Licania* sp. | -0.14 to  -1.42 | -0.35  to  -1.95 | -1.20 to  -2.30 | Powell et al. (2017) |
| Tropical rain forest in Tapajos National Forests, State of Pará, Brazil  (2°53’49.2”S, 54°57’07.2”W) | Throughfall exclusion experiment (TFE) | *Inga* sp., *Eschweilera* sp., *Protium* sp., and *Licania* sp. | NA | | -1.10 to  -2.00 |  |
| Low seasonality forest: Cuieras Biological Reserve, Manaus, Amazonas, Brazil (2°61’S, 60°21’W) | Minimum Ψ was measured during the peak of the dry season of non-ENSO (left in Ψ column) and ENSO (right in Ψ column) years. | *Caryocar* sp., *Dypterix* sp., *Eschweilera* sp., *Goupia* sp., *Gustavia* sp., *Lecyths* sp., *Maquira* sp., *Minquartia* sp., *Ocotea* sp., *Pouteria* sp., *Pouteria* sp., *Protium* sp., *Scleronema* sp., and *Zygia* sp. | -0.57 to  -2.09 | -1.10  to  -2.89 | -1.01 to  -4.47 | Barros et al. (2019) |
| High seasonality forest: Tapajos National Forest, Santarem, Para, Brazil (2°51’S, 54°58’W) |  | *Amphyrrhox* sp., *Chamaecrista* sp., *Coussarea* sp., *Endopleura* sp., *Erisma* sp., *Manilkara* sp., *Mezilaurus* sp., *Miconia* sp., *Minquartia* sp., *Protium* sp., *Rinourea* sp., and *Tachigali* sp. | -1.06  to  -2.68 | -1.06  to  -4.43 | -1.52 to  -5.02 |  |
| Tropical rain forest in Caxiuana National Forest, State of Pará, Brazil  (1°43′S, 51°27′W) | Control | *Aspidosperma* sp., *Eschweilera* sp., *Inga* sp., *Licania* sp., *Micropholis* sp., *Minquartia* sp., *Pouteria* sp., *Protium* sp., *Swartzia* sp., *Syzygiopsis* sp., *Virola* sp., and *Vouacapoua* sp. | -1.30 to -2.40 | | -1.40 to  -3.10 | Bittencourt et al. (2020) |
|  | Through-fall exclusion (TFE) |  | -1.10 to -2.80 | | -1.10 to  -3.20 |  |
| Tropical rain forest in Daintree Rainforest Observatory Research Facility, Cape Tribulation, Queensland, Australia  (16°06’14.4”S, 145°26’56.4”E) | - | *Dysoxylum* sp., *Elaeocarpus* sp., and  *Syzygium* sp. | -1.23 to -1.62 | | -2.10 to  -3.06 | Nolf et al. (2015) |
| Tropical rain forest in New Caledonia, the north of the Tropic of Capricorn in the southwest Pacific Ocean  (21°30′S, 165°30′E) | - | *Amborella* sp., *Ascarina* sp., *Cryptocarya* sp., *Hedycarya* sp., *Hedycarya* sp., *Kibaropsis* sp., *Nemuaron* sp., *Paracryphia* sp., *Quintinia* sp., and *Zygogynum* sp. | NA | | -2.10 to  -4.00 | Trueba et al. (2017) |
| Xishuangbanna Tropical Botanical Garden, Yunnan Province, China  (21°54’N, 101°46’E) | Lianas | *Celastrus* sp., *Marsdenia* sp., *Ventilago* sp., and *Mucuna* sp. | NA | | -1.04 to  -1.57 | Chen et al. (2017) |
|  | Trees | *Celtis* sp., *Ficus* sp., *Harpullia* sp., *Michelia* sp., and *Streblus* sp. | NA | | -1.43 to  -2.93 |  |
| Tropical non-karst and karst forests in Xishuangbanna Tropical Botanical Garden, Yunnan Province, China  (21°54’N, 101°46’E) | Data collected in tropical non-karst forest | *Bauhinia* sp., *Bischofia* sp., *Castanopsis* sp., *Lagerstroemia* sp., *Millettia* sp., *Syzygium* sp., *Millettia* sp., *Uncaria* sp., and *Byttneria* sp. | NA | | -1.10 to  -2.00 | Zhu et al. (2017) |
|  | Data collected in tropical karst rain forest | *Alphonsea* sp., *Celtis* sp., *Cipadessa* sp., *Cleistanthus* sp., *Croton* sp., *Lasiococca* sp., *Pistacia* sp., *Turpinia* sp., *Combretum* sp., and *Ventilago* sp. | NA | | -1.30 to  -4.10 |  |
| Tropical karst forest in Xishuangbanna Tropical Botanical Garden, Yunnan Province, China  (21°54’N, 101°46’E) | Minimum Ψ was measured in normal dry season (left in Ψ column) and extreme dry season (right in Ψ column). | Evergreen  *Alphonsea* sp., *Celtis* sp., *Cleidion* sp., *Cleistanthus* sp., *Pistacia* sp., *Lasiococca* sp., and *Turpinia* sp.  Brevi-deciduous  *Cipadessa* sp., *Croton* sp., *Ficus* sp., *Lagerstroemia* sp., *Mayodendron* sp.  Liana  *Bauhinia* sp., *Combretum* sp., *Gnetum* sp., *Ventilago* sp. | -0.98  to  -2.41  -1.34  to  -1.80  -0.60  to  -1.60 | -2.13  to  -6.55  -1.10  to  -2.68  -0.65  to  -2.95 | -1.96 to -4.12  -0.80 to  -2.40  -1.17 to  -2.06 | Tan et al. (2020) |
| Seasonal evergreen forest Khao Yai National Park, Thailand  (14°26’N, 101°22’E) | Midday leaf Ψ was measured in wet season (left in Ψ column) and dry season (right in Ψ column).  P_50_ was done once throughout the study. | Old-growth forest  *Dipterocarpus* sp.*, Sloanea* sp.*, Ilex* sp.*, Symplocos* sp., and *Schima* sp.  Intermediate forest  *Schima* sp.*, Machilus* sp.*, Eurya* sp.*, Symplocos* sp.*,* and *Syzygium* sp.  Young forest  *Cratoxylum* sp.*, Syzygium* sp.*, Adinandra* sp.*, Syzygium* sp.*,* and *Symplocos* sp. | -0.60  to  -1.08  -0.72  to  -1.01  -0.80  to  -1.46 | -0.43  to  -1.21  -0.64  to  -1.68  -0.57  to  -2.54 | -0.89  to  -4.17  -2.30 to  -4.01  -2.24 to  -5.97 | This study |

Table S2 Maximum vessel length (MVL) of selected dominant tree species from different successions in Khao Yai National Park, Thailand. Values are means ± one standard deviation with sample size of 3.

| Species | MVL (cm) |
| --- | --- |
| Old-growth forest (OF) | **34.90 ± 12.90** |
| *Dipterocarpus gracilis* | 50.33 ± 4.16 |
| *Sloanea sigun* | 27.33 ± 2.08 |
| *Ilex chevalieri* | 15.33 ± 1.53 |
| *Symplocos cochinchinensis* | 42.00 ± 3.00 |
| *Schima wallichii* | 39.70 ± 1.53 |
| Intermediate forest (IF) | **43.30 ± 4.54** |
| *Schima wallichii* | 46.33 ± 2.08 |
| *Machilus gamblei* | 45.67 ± 6.43 |
| *Eurya acuminata* | 41.33 ± 3.79 |
| *Symplocos cochinchinensis* | 44.00 ± 3.00 |
| *Syzygium nervosum* | 39.00 ± 4.36 |
| Young forest (YF) | **38.40 ± 2.95** |
| *Cratoxylum cochinchinense* | 40.00 ± 3.61 |
| *Syzygium antisepticum* | 39.00 ± 3.00 |
| *Adinandra integerrima* | 37.67 ± 3.79 |
| *Syzygium nervosum* | 36.33 ± 3.06 |
| *Symplocos cochinchinensis* | 39.00 ± 1.73 |

Reference

Barros, F. D., Bittencourt, P. R. L., Brum, M., Restrepo-Coupe, N., . . . Oliveira, R. S. (2019). Hydraulic traits explain differential responses of Amazonian forests to the 2015 El Nino-induced drought. *New Phytologist*, *223*(3), 1253-1266. https://doi.org/10.1111/nph.15909

Bittencourt, P. R. L., Oliveira, R. S., da Costa, A. C. L., Giles, A. L., . . . Rowland, L. (2020). Amazonia trees have limited capacity to acclimate plant hydraulic properties in response to long-term drought. *Global Change Biology*, *26*(6), 3569-3584. https://doi.org/https://doi.org/10.1111/gcb.15040

Chen, Y. J., Schnitzer, S. A., Zhang, Y. J., Fan, Z. X., . . . Cao, K. F. (2017). Physiological regulation and efficient xylem water transport regulate diurnal water and carbon balances of tropical lianas. *Functional Ecology*, *31*(2), 306-317. https://doi.org/10.1111/1365-2435.12724

Nolf, M., Creek, D., Duursma, R., Holtum, J., . . . Choat, B. (2015). Stem and leaf hydraulic properties are finely coordinated in three tropical rain forest tree species. *Plant Cell and Environment*, *38*(12), 2652-2661. https://doi.org/10.1111/pce.12581

Powell, T. L., Wheeler, J. K., de Oliveira, A. A. R., da Costa, A. C. L., . . . Moorcroft, P. R. (2017). Differences in xylem and leaf hydraulic traits explain differences in drought tolerance among mature Amazon rainforest trees. *Global Change Biology*, *23*(10), 4280-4293. https://doi.org/10.1111/gcb.13731

Rowland, L., da Costa, A. C. L., Galbraith, D. R., Oliveira, R. S., . . . Meir, P. (2015). Death from drought in tropical forests is triggered by hydraulics not carbon starvation. *Nature*, *528*(7580), 119-122. https://doi.org/10.1038/nature15539

Tan, F. S., Song, H. Q., Fu, P. L., Chen, Y. J., . . . Zhu, S. D. (2020). Hydraulic safety margins of co-occurring woody plants in a tropical karst forest experiencing frequent extreme droughts. *Agricultural and Forest Meteorology*, *292*. https://doi.org/ARTN 10810710.1016/j.agrformet.2020.108107

Trueba, S., Pouteau, R., Lens, F., Feild, T. S., . . . Delzon, S. (2017). Vulnerability to xylem embolism as a major correlate of the environmental distribution of rain forest species on a tropical island. *Plant Cell and Environment*, *40*(2), 277-289. https://doi.org/10.1111/pce.12859

Zhu, S. D., Chen, Y. J., Fu, P. L., & Cao, K. F. (2017). Different hydraulic traits of woody plants from tropical forests with contrasting soil water availability. *Tree Physiology*, *37*(11), 1469-1477. https://doi.org/10.1093/treephys/tpx094
